# Associations between age and cortisol awakening response in patients with borderline personality disorder

**DOI:** 10.1007/s00702-021-02402-3

**Published:** 2021-08-14

**Authors:** Juliane Rausch, Elisa Flach, Angelika Panizza, Romuald Brunner, Sabine C. Herpertz, Michael Kaess, Katja Bertsch

**Affiliations:** 1 Department of General Psychiatry, Center for Psychosocial Medicine, Heidelberg University, Heidelberg, Germany; 2 Department of Child and Adolescent Psychiatry and Psychotherapy, Central Institute of Mental Health, Medical Faculty Mannheim, University of Heidelberg, J5, 68159 Mannheim, Germany; 3 Department of Child and Adolescent Psychiatry, Centre for Psychosocial Medicine, Heidelberg University, Heidelberg, Germany; 4 Department of Psychosomatic Medicine and Psychotherapy, Central Institute of Mental Health, Medical Faculty Mannheim, Heidelberg University, Heidelberg, Germany; 5 Clinic of Child and Adolescent Psychiatry, Psychosomatics and Psychotherapy, University of Regensburg, Regensburg, Germany; 6 University Hospital of Child and Adolescent Psychiatry and Psychotherapy, University of Bern, Bern, Switzerland; 7 Department of Psychology, Ludwig-Maximilians-University Munich, Munich, Germany

**Keywords:** Borderline personality disorder, Aging, Stress, Hypothalamic–pituitary–adrenal axis, Cortisol awakening response

## Abstract

Patients with borderline personality disorder (BPD) often display increased stress vulnerability, which may be linked to altered hypothalamus–pituitary–adrenal (HPA) axis functioning. Corresponding deviations of the cortisol awakening response (CAR) are presumed to mirror maladaptive neuroendocrine processes, which may explain why CARs are increased compared to healthy controls (HC). Prior research speculated that these alterations may be caused by early life stress and/or chronic stress related to the ongoing burden of the disorder. Yet, it remains to be investigated how BPD influences CAR in the course of development. Therefore, the current study examined CAR in female adolescents and adults with BPD compared to HC with a particular focus on associations with age. These potential associations were especially focused, as it was hypothesized that the CAR would be even more elevated (i.e., higher) in older individuals with BPD. CAR was assessed in 54 female individuals with BPD (aged 15–40 years) and 54 sex-, age-, and intelligence-matched HC (aged 15–48 years). Group differences were investigated and analyses of covariance using age as continuous predictor were performed to analyze potential developmental associations with CAR alongside BPD-specific effects. Pearson’s correlations were calculated to examine associations between CAR and age. Analyses were repeated with potential confounders as control factors. Results not only demonstrated increased CARs in female individuals with BPD compared to HC but demonstrated elevated CARs with increasing age in BPD individuals exclusively. Effects remained stable after controlling for potential confounders. Thereby, findings suggest that endocrine alterations in BPD may reinforce with increasing age and BPD chronicity.

## Introduction

Borderline personality disorder (BPD) is a severe and chronic mental disorder, which has been defined by emotion dysregulation, impulsivity, identity disturbance, and interpersonal problems (American Psychiatric Association 2013). Several BPD symptoms, such as inappropriate and intense anger, stress-associated dissociation or self-harm, are linked to altered stress vulnerability and can possibly be attributed to changes in hypothalamus–pituitary–adrenal (HPA) axis functioning, which is one of the major endocrine stress systems. A common marker of the HPA axis is the cortisol awakening response (CAR), which usually rises steeply in the morning after awakening and proceeds according to a diurnal rhythm (Edwards et al. 2001; Pruessner et al. 1997). Deviations from the typical CAR pattern due to psychosocial, psychiatric and health-related parameters, such as psychosocial stress, are presumed to mirror maladaptive neuroendocrine processes (Clow et al. 2004; Schmidt-Reinwald et al. 1999).

In general, HPA axis functioning depends on multiple factors and age might be a particularly important one. From a developmental perspective, HPA axis functioning is highly reactive after birth. In childhood, however, the HPA axis can become hyporesponsive to stress (Gunnar and Quevedo 2007). In puberty, HPA axis markers reach levels similar to those in adulthood, though it has been assumed that the HPA axis is ‘reprogrammed’ based on individual experiences of emotional stress (Quevedo et al. 2012). Besides, existing research suggests that basal cortisol secretion increases with age across the lifespan (Seeman et al. 2001) and that HPA axis dysfunctions may contribute to aging-related diseases such as cognitive deficits in some individuals (Gupta and Morley 2014). Until now, however, little research focused directly on links between age and HPA axis functioning in mental disorders.

Interestingly, existing research further suggests that mental disorders can be characterized by “accelerated aging”, which means that certain biological correlates amplify with increasing illness duration. For instance, Wolkowitz et al. (2010) highlighted that in depression, stronger alterations of HPA axis functioning over time are accompanied by increased cell damaging processes, immune dysregulation, and oxidative stress. Similarly, Miller and Sadeh (2014) reviewed existing evidence on the “accelerated aging” hypothesis in post-traumatic stress disorder (PTSD) and reported that chronic and repeated activation of the HPA axis—for example, when re-experiencing traumatic experiences—has deleterious effects on the brain, such as hippocampal atrophy and neuronal cell death. In short, altered HPA axis functioning may be related to chronification of mental disorders and it seems likely that comparable links exist between aging and HPA axis functioning in BPD. Yet, the time course between HPA axis functioning and aging needs to be examined in greater detail, in particular for patients with BPD.

With regard to BPD in particular, research demonstrated increased CARs in adult patients (Lieb et al. 2004; Rausch et al. 2015), which can possibly be attributed to individuals with BPD experiencing more daily hassles and inner tension during the day (Jovev and Jackson 2006). Further, it has been shown that individuals with BPD with histories of childhood abuse are at high risk for revictimization as adults, and this might play an important role in maintenance or chronification of stress vulnerability (Paris and Zweig-Frank 1992). For adolescents, previous research revealed that CARs were higher in individuals with histories of childhood maltreatment and pronounced BPD symptoms than in individuals with childhood maltreatment but without BPD symptoms (Kaess et al. 2017). Compared to healthy controls (HC), CARs were also increased in adolescents engaging in non-suicidal self-injury (NSSI), which is an important precursor of BPD development (Reichl et al. 2016). Taken together, previous findings suggest that BPD pathology is related to HPA axis alterations (i.e., increased CAR), though it has not been investigated whether this association varies for different age groups.

Based on existing research, the present study, therefore, investigated if individuals with BPD display elevated CARs, even when a broader age range is examined than in previous studies. Correspondingly, we hypothesized that individuals with BPD would display an elevated CAR compared to healthy controls. Moreover, associations between age and CAR in individuals with BPD were examined in particular. Owing to the notion that BPD can be seen as a life span disorder and frequently chronifies due to frequent revictimization, daily hassles and other stressful experiences, we hypothesized that individuals with BPD would display an even more increased CAR with older age.

## Methods

### Participants

Participants were 54 female individuals with a current diagnosis of BPD according to the diagnostic criteria of DSM-IV (BPD; *M*_age_ = 23.7 years, SD = 6.5, range: 15–40 years) and 54 female age-, and intelligence-matched^1^ healthy controls (HC; *M*_age_ = 23.0, SD = 7.4, range: 15–48 years), who had never received a psychiatric diagnosis or undergone any psychological or psychiatric treatment (see Table 1 for details on sample characteristics including comorbid disorders). Participants were recruited through the resident’s registration office, advertisements and clinical referral from in- and outpatient units. General exclusion criteria comprised current substance abuse (urine screening), substance abuse in the past two months (interview), and severe medical illness. Individuals with BPD were additionally excluded when presenting with a lifetime diagnosis of schizophrenia, schizoaffective or bipolar disorder, or substance dependence in the past year. The study was part of the KFO-256 (Schmahl et al. 2014), a German consortium on mechanisms underlying emotion dysregulation in BPD.^2^ Sensitivity analyses indicated that the included sample was large enough to detect small group differences of *d* ≥ 0.58 in AUC_G_ of the CAR as reported in our former study (Rausch et al. 2015) with a power of 1-ß ≥ 0.80. Data partly overlap with data published by Rausch et al. (2015); however, the current research question has not been addressed by a previous publication by the consortium. The Ethics Committee at Heidelberg University approved the study. Participants signed written informed consent and received a financial compensation. Written informed consent was also obtained from all parents or legal guardians if they were minors.

**Table 1:** Demographic and clinical data of patients with borderline personality disorder (BPD) and healthy controls (HC)

	BPD patients [*M* ± SD; *n* (%)]	Range	Healthy controls [*M* ± SD; *n* (%)]	Range	*t*/χ^2^	*p*
Age (years)	23.7 ± 6.5	[15, 40]	23.0 ± 7.4	[15, 48]	0.51	0.611
Body Mass Index (kg/m^2^)	23.5 ± 5.3	[18.59, 38.30]	22.9 ± 4.1	[18.34, 37.23]	1.57	0.120
Smokers (*n*)	20 (37%)		8 (15%)		6.94	0.008
Oral contraceptive use (*n*)	12 (22%)		27 (50%)		9.03	0.003
Regular medication^a^	4 (7%)		0		4.15	0.042
Time of awakening	0702 h ± 1.5 h		0747 h ± 1.4 h		2.46	0.120
Sleep duration (hours)	7.9 ± 1.3	[4, 10]	8.1 ± 1.1	[5.5, 10.75]	− 0.98	0.332
Childhood traumatization (CTQ Total Score)	56.6 ± 18.1	[25, 116]	31.6 ± 10.8	[25, 81]	8.39	< 0.001
Depressiveness (BDI-II Score)	26.7 ± 11.0	[0, 52]	3.1 ± 3.4	[0, 20]	14.52	< 0.001
Borderline symptom severity (BSL-23 Score)	2.1 ± 0.9	[0, 3.65]	0.1 ± 0.1	[0, 0.65]	16.14	< 0.001
No. of DSM-IV BPD criteria	6.3 ± 1.5	[5, 45]	0	[0, 3]		
Current major depression (*n*)	14 (26%)		0			
Lifetime major depression (*n*)	39 (72%)		0			
Current post-traumatic stress disorder (*n*)	12 (22%)		0			
Lifetime post-traumatic stress disorder (*n*)	14 (26%)		0			

*M *mean, *SD *standard deviation, *BSL-23* borderline symptom list, *BDI-II* Beck Depression Inventory-II, *CTQ* Childhood Trauma Questionnaire

^a^One patient was treated with Fluoxetine, one with Fluoxetine and Lorazepam, one with Fluoxetine and Fenofibrate, and one with Agomelatine

### Measures

Axis I disorders were assessed by qualified diagnosticians using the Structured Clinical Interview for DSM-IV (SCID-I; First and Gibbon 2004). BPD, avoidant PD and antisocial PD were examined using the International Personality Disorder Examination (IPDE; Loranger et al. 1994). Interviews were performed by experienced diagnosticians, who had at least a master’s degree in psychology or medical doctorate and underwent standardized training resulting in high interrater reliability (ICC $\ge$ 0.091 for the number of BPD criteria). Additionally, BPD symptom severity was examined using the short version of the Borderline Symptom List (BSL-23; Bohus et al. 2009), depressiveness using the Beck Depression Inventory (BDI-II; Beck and Steer 1984), and history of childhood traumatization using the Childhood Trauma Questionnaire (CTQ; Bernstein et al. 1994). Demographic information was assessed using standardized questionnaires. Height and weight were measured at the day of diagnostic interview.

### Cortisol assessment and analysis

#### Cortisol awakening response

Cortisol awakening response (CAR) was assessed on two consecutive weekdays to get a reliable measure of basal HPA axis activity (Hellhammer et al. 2007). At both days, participants collected saliva samples with salivette devices (Sarstedt, Rommelsdorf, Germany) at home at awakening and 30, 45, and 60 min later by gently chewing on a cotton swab for about 1 min. Saliva sample collection was protocolled and time-locked with electronic monitoring systems [Medication Event Monitoring System (MEMS^®^)], which are known to enhance compliance of participants (Kudielka et al. 2012). During sampling periods, participants were instructed to refrain from drinking anything but water, brushing their teeth, eating, and exercising. Samples were stored in refrigerators or freezers until storage in the laboratory and frozen at − 20 °C until biochemical analysis. Cortisol concentration was measured using a commercially available chemiluminescence immunoassay (CLIA) with high sensitivity of 0.16 ng/ml (IBL) and intra- and interassay coefficients of less than 6% and 8%, respectively. Area under the curve with respect to the ground (AUC_G_) and mean cortisol increase (MnInc) were computed to estimate trait measures of HPA axis activity.^3^ Data were not logarithmized and no participants had to be excluded due to extreme outliers.

#### Statistical analyses

Statistical analyses were performed with IBM SPSS 22 with *α* set to 0.05. Table 1 shows demographic and self-report data of BPD individuals and HC based on comparisons using *t*-tests for independent groups and *χ*^2^-tests. Group differences and age effects in cortisol data were examined using a mixed-design analysis of covariance (ANCOVA) with the between-subject factor group (BPD vs. HC), the within-subject factor time after awakening (0, + 30, + 45, + 60 min) and the continuous predictor age. Additional ANCOVAs examining group and age were calculated for AUC_G_ and MnInc of the CAR. Dunn’s multiple comparisons including Bonferroni corrections were used post hoc to examine significant effects of time or group by time; Pearson’s correlations were used for analyses examining associations with age. Where appropriate, the Huynh–Feldt procedure was applied to correct for violations of the sphericity assumption. Correlation coefficients were compared using Fisher’s *Z*-transformation. ANCOVAs were repeated with potential confounders as control factors [smoking, contraceptive use, medication use, current diagnosis of major depression or PTSD, childhood trauma (CTQ sum scores)]. Effect sizes are reported as proportion of explained variance [partial eta squared (*η*^2^)].

## Results

**Fig. 1:**
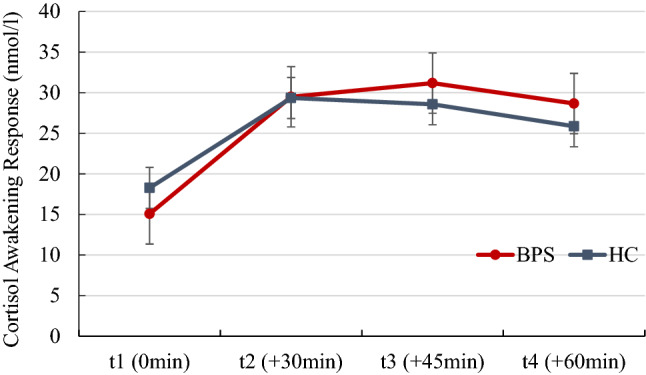
Cortisol awakening response of patients with borderline personality disorder (BPD) and healthy controls (HC). Standard error is depicted

Descriptively, there was an inverted U-shaped cortisol response after awakening across participants as well as higher cortisol levels in individuals with BPD than in HC across time points (Fig. 1). In line with this, the repeated-measures ANCOVA revealed a significant main effect of time point, [*F*(3, 312) = 4.70, *p* = 0.011, *η*^2^ = 0.04] with a significant rise from *t*1 (awakening) to *t*2 (+ 30 min, *p* < 0.01), *t*3 (+ 45 min, *p* > 0.01), and *t*4 (+ 60 min, *p* < 0.01) and a significant drop from *t*3 (+ 45 min) to *t*4 (+ 60 min, *p* < 0.05). Furthermore, individuals with BPD tended to show higher cortisol levels than HC in general although this effect was statistically not significant [*F*(1,104) = 3.37, *p* = 0.069, *η*^2^ = 0.03; Fig. 1].

The group by age interaction [*F*(1,104) = 3.91, *p* = 0.051, *η*^2^ = 0.04] as well as the group by age by time point interaction [*F*(3,312) = 3.65, *p* = 0.029, *η*^2^ = 0.03] suggested differential associations between age and cortisol levels in individuals with BPD and HC. In fact, age was positively associated with cortisol levels in individuals with BPD (*t*1: *r* = 0.12, *p* = 0.394, *t*2: *r* = 0.30, *p* = 0.030, *t*3: *r* = 0.27, *p* = 0.049, *t*4: *r* = 0.23, *p* = 0.095), but not in HC (*t*1: *r* = 0.06, *p* = 0.682, *t*2: *r* = -0.08, *p* = 0.574, *t*3: *r* = − 0.14, *p* = 0.323, *t*4 = *r* = − 0.13, *p* = 0.344) with significant group differences in correlation coefficients at *t*2 (*z* = 1.97, *p* = 0.025), *t*3 (*z* = 2.11, *p* = 0.017), and *t*4 (*z* = 1.84, *p* = 0.033).

**Fig. 2:**
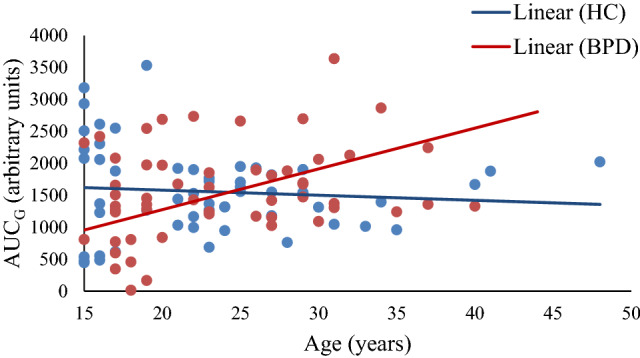
Association between area under the curve with respect to ground (AUC_G_) of the cortisol awakening response and age in patients with borderline personality disorder (BPD) and healthy controls (HC)

The mean cortisol levels of individuals with BPD and of HC at the different measurement time points after awakening as well as the MnInc of the CAR and the mean AUCG of the CAR of both groups are depicted in Table 2. While analyses failed to reach statistical significance, AUC_G_ data revealed a similar pattern of results as patients tended to show higher AUC_G_ [*F*(1,104) = 3.51, *p* = 0.064, *η*^2^ = 0.03] and a differential association between AUC_G_ and age than HC [*F*(1,104) = 3.88, *p* = 0.051, *η*^2^ = 0.04]. AUC_G_ and age were positively correlated in individuals with BPD (*r* = 0.28, *p* = 0.044), but not in HC (*r* = − 0.09, *p* = 0.536; group comparison: *z* = 1.91, *p* = 0.028; Fig. 2).

For MnInc, the main effect of group was not statistically significant, [*F*(1,104) = 2.53, *p* = 0.115, *η*^2^ = 0.02]; however, post hoc tests of the group by age interaction [*F*(1,104) = 5.54, *p* = 0.020, *η*^2^ = 0.05] showed a positive correlation between age and MnInc in individuals with BPD (*r* = 0.24, *p* = 0.081), but not in HC (*r* = − 0.09, *p* = 0.536, group comparison: *z* = 1.69, *p* = 0.045).

**Table 2:** Mean cortisol levels at awakening (0 min; *t*1) as well as + 30 min (*t*2), + 45 min (*t*3), and + 60 min (*t*4) after awakening (in nmol/l), area under the curve with respect to ground (AUC_G_), and mean increase (MnInc) of the Cortisol awakening response of patients with borderline personality disorder (BPD) and healthy controls (HC)

	BPD patients (*M* ± SD)	Healthy controls (*M* ± SD)
*t*1 (0 min)	15.1 ± 8.2	18.3 ± 8.2
*t*2 (+ 30 min)	29.5 ± 14.5	29.3 ± 13.6
*t*3 (+ 45 min)	31.2 ± 15.1	28.6 ± 14.6
*t*4 (+ 60 min)	28.7 ± 15.0	25.9 ± 12.4
AUC_G_	1572.0 ± 720.8	1556.8 ± 684.8
MnInc	14.7 ± 12.3	9.6 ± 9.9

Taken together, there was a trend for elevated cortisol awakening responses in individuals with BPD compared to HC, but this effect was qualified by age with greater differences between the BPD and HC group with increasing age. Effects remained stable after controlling for potential confounding effects of medication use, contraceptive use, smoking, current diagnosis of major depression and PTSD as well as childhood trauma (CTQ).

## Discussion

This is, to our knowledge, the first study focusing on age-specific endocrine alterations in BPD. Our findings indicate that female individuals with BPD of different ages tend to show increased CARs compared to healthy female controls, which coincides with earlier research on increased salivary cortisol responses to awakening (Carvalho Fernando et al. 2012) and elevated salivary cortisol levels over the day (Lieb et al. 2004). Interestingly, our findings further show that endocrine differences between experimental groups increase with age of individuals, independent of potential confounders. Thus, CARs rose with age of individuals in the BPD group, while staying on a constant level in the control group. Since BPD commonly develops during childhood and adolescence, this could indicate that the duration of BPD symptoms is associated with increasing HPA axis dysfunction and related stress vulnerability. Consequently, a larger CAR with increasing age may be seen as a correlate of BPD chronicity, considering that individuals with BPD report more frequent and intense daily hassles (Jovev and Jackson 2006), display elevated levels of stress associated with inner tension (Kuo and Linehan 2009), and are revictimized more frequently (Paris and Zweig-Frank 1992) over the life course. Consequently, less attenuation of the CAR in older individuals with BPD may be viewed as a neuroendocrine correlate of a long stress-related illness.

Patients with BPD reported significantly more childhood trauma than healthy controls, which is consistent with previous reports on adverse childhood experiences in this patient population (e.g. Infurna et al. 2016). Yet, statistical analyses indicated that childhood trauma did not confound the association between experimental group and CAR, which coincides with earlier research showing only weak positive associations between self-reported childhood trauma and basal saliva cortisol levels in BPD (Carvalho Fernando et al. 2012), partially replicatin﻿﻿g own results (Rausch et al. 2015). The current study also matches with research by Reichl et al. (2016), who demonstrated that childhood adversity differentially affected diurnal slope of the CAR in NSSI patients and healthy controls.

To examine this issue in greater detail, future studies should use longitudinal designs to explicitly investigate associations between age, stress responsivity and altered HPA axis functioning in individuals with BPD over time. A better understanding of age-related diseases seems further crucial as the HPA axis regulates homeostasis, for instance within the cardiovascular, neuroendocrine, metabolic or immune system. Future research may, therefore, investigate whether altered HPA axis functioning in individuals with BPD is related to lower physical health (Powers and Oltmanns 2012), i.e., cognitive deficits (Gupta and Morley 2014), Type II diabetes (Frankenburg and Zanarini 2006) or stroke (Chen et al. 2017). This seems particularly relevant given that individuals tend to be at risk for severe physical illness and subsequent mortality (Fok et al. 2014). Finally, future research may also investigate whether alterations of the CAR can be normalized by early treatment of BPD. By way of example, research may investigate if successful treatment leads to a normalization of the HPA axis and whether such a normalization is associated with improved physical and psychological health in general.

When interpreting the current findings, several limitations and strengths of the current study should be considered. First, the study applied a cross-sectional design, which has limited explanatory power given that long-range hormonal changes were investigated. Subsequently, conclusions on associations between age and endocrine functioning in patients with BPD should be interpreted cautiously and require careful replication using longitudinal research. To explore the development of altered HPA axis functioning due to BPD chronicity, further studies may investigate endocrine alterations in patients with BPD of different ages and including dynamic parameters of HPA axis responsiveness such as psychosocial challenges, such as the Trier Social Stress Test (Kirschbaum et al. 1993), or pharmacological challenges, such as the dexamethasone suppression test, which potentially enables a differentiated profile of HPA axis functioning in individuals with BPD. To date, meta-analytic research indicated that especially psychosocial challenges and continuous cortisol measures—such as the CAR—are altered in individuals with BPD (Drews et al. 2019). Here, cortisol profiles seem to be blunted in response to psychosocial challenges but elevated based on continuous measures when using healthy individuals as comparison. Furthermore, the experimental design rendered inclusion of male individuals with BPD impossible, so that the current findings may not be generalized to male patients with BPD (Seeman et al. 2001). Since only females were included for the current sample, it needs to be mentioned that menstrual cycle has not been assessed systematically. We can, therefore, not exclude the possibility that menstrual cycle influenced associations between CAR and psychopathology. Yet, existing studies indicate that menstrual cycle does not significantly affect such an association (Kirschbaum et al. 1999; Kudielka et al. 2003; Wust et al. 2000). This notion is further supported by a recent meta-analysis on HPA axis functioning in individuals with BPD, which could not find any significant gender differences using meta-regressions (Drews et al. 2019) Nonetheless, future studies may systematically assess and analyze menstrual cycle to examine the influence of this variable more closely. With regard to strengths of the current study, this is one of the few studies covering a wide age range in adults and adolescents with BPD, which allowed for a thorough investigation of associations of age and HPA axis activity. Moreover, the reliable ambulatory assessment enabled precise and comprehensive measurements and analyses were controlled for a broad range of potential confounders.

Taken together, the present study not only suggests differential CAR levels in individuals with BPD and healthy controls but further indicates close links between aging and HPA axis dysfunction in individuals with BPD. Possibly, these alterations are related to enhanced anticipation of upcoming stressors with increasing age, which in turn, indicates an important connection with BPD chronicity. Additionally, altered CAR levels may have far-reaching consequences for several physical health conditions. To further investigate this issue, future studies should not only include larger adolescent samples and apply longitudinal designs but may additionally focus on whether preventive interventions translate into alterations of the neuroendocrine stress response in individuals with BPD.

## Footnotes

1 Raven’s Progressive Matrices were used an estimate for intelligence (John and Raven 2003).

2 Projects by the KFO include participants recruited by a common recruitment unit with psychometric data of all participants being monitored in a central data bank. Samples across KFO-256 studies may show overlap in participants.

3 AUC_G_, which represents the entire area under the cortisol awakening response with respect to the ground, was calculated according to a formula described by Pruessner et al. (2003). MnInc, which represents cortisol increase after awakening, was calculated with a formula provided by Wust et al (2000). AUC_G_ and MnInc were calculated for each participant and day and then averaged over 2 days to form reliable indicators of overall cortisol secretory activity and dynamic cortisol responses following awakening.
